# Brucellosis in Dogs and Public Health Risk

**DOI:** 10.3201/eid2408.171171

**Published:** 2018-08

**Authors:** Martha E. Hensel, Maria Negron, Angela M. Arenas-Gamboa

**Affiliations:** Texas A&M University, College Station, Texas, USA (M.E. Hensel, A.M. Arenas-Gamboa);; Centers for Disease Control and Prevention, Atlanta, Georgia, USA (M. Negron)

**Keywords:** *Brucella canis*, serology, public health, dogs, brucellosis, bacteria, zoonoses

## Abstract

*Brucella canis* infects dogs and humans. In dogs, it can cause reproductive failure; in humans, it can cause fever, chills, malaise, peripheral lymphadenomegaly, and splenomegaly. *B. canis* infection in dogs is underrecognized. After evaluating serologic data, transmission patterns, and regulations in the context of brucellosis in dogs as an underrecognized zoonosis, we concluded that brucellosis in dogs remains endemic to many parts of the world and will probably remain a threat to human health and animal welfare unless stronger intervention measures are implemented. A first step for limiting disease spread would be implementation of mandatory testing of dogs before interstate or international movement.

*Brucella canis* is a gram-negative coccobacillary bacterium that primarily causes reproductive failure in dogs (*1*). The genus *Brucella* comprises 12 recognized species (*2*). Of these, *B. melitensis*, *B.* *abortus*, and *B.* *suis* are well-known causes of undulant fever and influenza-like symptoms in humans, but *B. canis* is less recognized as the cause of a zoonosis (*3*). In this review, we highlight information regarding occurrence of brucellosis in dogs, emphasizing *B. canis* as an underrecognized pathogen and describing current knowledge about its zoonotic potential.

## Epidemiology

*B. canis* was initially characterized in 1966 after several outbreaks of abortion and infertility in dogs in multiple states (*1*). Since the discovery of *B. canis* as a cause of abortion, outbreaks in breeding and research kennels have been sporadically reported worldwide (*4*–*7*). The primary hosts are domesticated dogs; however, *B. canis* in wild canids and humans has also been reported (*8*,*9*).

Brucellosis in dogs occurs worldwide and is endemic to the Americas, Asia, and Africa (Figure) (*10*). In the 1970s and early 1980s, serologic surveys of dogs from multiple countries demonstrated a wide range of seropositivity, from 1% to 28%, depending on the country (Technical Appendix). Within the past 30 years, few studies have been conducted to evaluate disease occurrence and distribution in the United States, so the current status is unknown. However, in the past 2 decades, serologic studies of dogs have been published from countries in Africa, Asia, and South America and have reported moderate to high seroprevalence, ranging from 6% to ≈35% (Technical Appendix). This wide range of seroprevalence could be attributed to multiple factors, including but not limited to true disease prevalence in the region or country, sampling design and study sample, and diagnostic test algorithm used.

**Figure:**
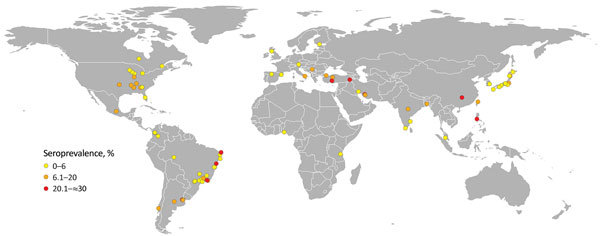
Locations of published *Brucella canis* serologic surveys of dogs (online Technical Appendix, https://wwwnc.cdc.gov/EID/article/24/8/17-1171-Techapp1.pdf). Each dot represents 1 published study; colors represent seroprevalence determined in each study. Cartography: Cecilia Smith.

*B. canis* infection in dogs occurs predominantly through ingestion, inhalation, or contact with aborted fetuses or placenta, vaginal secretions, or semen (*11*,*12*). Like the rest of the *Brucella* species, *B. canis* exhibits tropism for reproductive tissue. Thus, infected dogs intermittently shed low concentrations of bacteria in seminal fluids and nonestrus vaginal secretions. Postabortion vaginal fluids contain a high level of bacteria and are a source of infection for other dogs and humans (*11*). Even after castration, dogs may still serve as a source of infection because the bacteria can persist in the prostate and lymphoid tissues (*13*,*14*). In addition to in reproductive secretions, dogs can shed the bacteria in the saliva, nasal secretions, and urine (*11*,*15*). Studies suggest that the concentration of *B. canis* in urine is higher in male than female dogs; this difference is attributed to urine contamination with seminal fluid (*11*). However, the role of urine as a source of infection is not fully understood.

## Clinical Manifestations in Dogs

The clinical signs of *B. canis* infection are not pathognomonic. Dogs may be subclinically affected or may exhibit signs of reproductive failure. In male dogs, *B. canis* causes epididymitis, prostatitis, and orchitis (*15*); chronic testicular and epididymal inflammation can lead to unilateral or bilateral testicular atrophy and infertility (*13*).

The typical manifestation in females is mid- to late-term abortion (during days 45–59), followed by an odorless, brown-to-yellow vaginal discharge for 1–6 weeks (*1*). Another manifestation is embryonic death with resorption, which appears as conception failure after an apparently successful mating (*1*). It is possible for an infected bitch to abort and subsequently have normal pregnancies or intermittently experience reproductive failure; these dogs may serve as reservoirs for infection in *B. canis*–naive dogs (*1*,*13*). Aborted pups have nonspecific lesions, such as subcutaneous edema, hemorrhage, or congestion (*1*). Pups from infected bitches that survive may be infected in utero or through nursing and can be bacteremic yet appear healthy (*13*). It is possible for seemingly healthy puppies from an infected bitch to disseminate the bacteria to other dogs and to humans (*16*). Because *B. canis* infection is the most common cause of reproductive failure in dogs, it should be ruled out before investigating other causes of infertility or abortion (*13*). However, if reproductive failure is not documented, canine brucellosis can be difficult to diagnose.

Another well-recognized manifestation of infection with *B. canis* is diskospondylitis, which can occur in otherwise healthy dogs or in those with a history of reproductive failure that was treated with antimicrobial drugs (*17*,*18*). Infected dogs have a history of lameness, spinal pain, neurologic dysfunction, muscle weakness, or any combination of these signs, caused by vertebral osteomyelitis and intervertebral disc infection (*18*). Incidence of diskospondylitis is higher in male than female dogs, perhaps because of a reservoir of bacteria in the prostate that results in intermittent bacteremia even in castrated males (*11*,*17*,*18*).

Antimicrobial drug treatment alone after signs of reproductive failure is usually unsuccessful because of the ability of the bacteria to sequester intracellularly for long periods and cause episodic bacteremia (*8*). The recommended course of treatment is multimodal and includes surgical sterilization and antimicrobial drugs.

## Diagnostic Testing in Dogs

### Serology

The initial diagnostic test for suspected brucellosis cases and the screening tool for evaluating breeding dogs is serologic testing (Table). Serologic tests evaluate antibody response against *Brucella* spp. cell wall antigens. *Brucella* spp. have 2 recognized cell wall morphologic appearances based on the structure of the *O*-polysaccharide subunit of lipopolysaccharide: smooth (considered more virulent; includes *B. abortus*, *B. suis*, and *B. melitensis*) and rough (*B. canis* and *B. ovis*) (*25*). These differences are noteworthy because serologic tests designed to detect infections with smooth *Brucella* spp. will not detect infection with *B. canis*.

**Table:** Diagnostic tests for *Brucella canis* in dogs*

Test type	Antigen detected or target DNA	Sensitivity, %	Specificity, %	Reference
Serologic				
Rapid slide agglutination	Cell wall	50–75	83.34–99.7	(*19*)
2-mercaptoethanol rapid slide agglutination	Cell wall	31.76–70	100	(*19*)
Agar-gel immunodiffusion, cell wall antigen	LPS, outer membrane protein	27.98–52.94	100	(*19*)
ELISA	LPS or CPAg	88–97	94.3–96.7	(*20*)
Immunochromatographic	R-LPS with outer membrane proteins	89.58	100	(*21,22*)
Other				
PCR (ITS66 and ITS279)	16S-23S rRNA gene	100	86.45–100	(*23*)
PCR (JPF/JPR)	Outer membrane protein 2	16.67 (whole blood); 92.31 (vaginal swab sample)	100 (whole blood); 51.92 (vaginal swab sample)	(*24*)

*CPAg, cytoplasmic protein antigen; JPF, forward primer; JPR, reverse primer; LPS, lipopolysaccharide; R-LPS, rough LPS.

The serologic methods most commonly used to screen for *B. canis* infections are the rapid slide agglutination test, 2-mercaptoethanol rapid slide agglutination test, agar-gel immunodiffusion, and ELISA (*8*). To confirm the results of these screening serologic methods, most diagnostic laboratories use the indirect fluorescent antibody test.

Use of serologic tests to diagnose *B. canis* infection has several pitfalls. The lack of a sensitive and specific screening test hampers the ability of veterinarians to diagnose the disease accurately. These tests are better at detecting early infections but have diminished sensitivity in chronically infected animals, which may be only intermittently bacteremic (*19*). Using *B. canis* M– antigen instead of *B. ovis* antigen reduces nonspecific reactions to the cell wall antigens of other gram-negative bacteria (e.g., *Pseudomonas* spp., *Actinobacillus equuli*, *Bordetella bronchiseptica*) and gram-positive bacteria (e.g., *Staphylococcus aureus*, *S. epidermidis*) and improves specificity (*14*,*26*). Furthermore, treating serum with 2-mercapthoethanol increases the specificity of the test by destroying IgM pentamers that can interfere with evaluation of IgG but does not fully eliminate false positives because of heterologous cross-reactions (*14*,*27*). Treatment with antimicrobial drugs can affect testing by eliminating bacteremia (*8*).

### Culture

The standard test for *B. canis* is culture (*8*). Commonly collected samples include blood, vaginal discharge, and semen. Of these, blood is the most commonly collected; however, because bacteremia can be intermittent, positive animals may be missed (*10*,*19*). The best time for culturing *Brucella* is 2–4 weeks after infection, after demonstration of reproductive failure, when bacteremia is the highest (*8*,*10*,*26*). Culture is not recommended if the dog has received antimicrobial drugs because they will clear the bacteremia regardless of the resolution of systemic disease (*8*). Culture requires up to 9 days, increasing the risk for exposure of laboratory personnel if the cultures are not handled appropriately (*28*).

### PCR

Several PCR primers have been designed to detect *B. canis* DNA in whole blood, vaginal secretions, and semen. PCR has the potential as a rapid, discriminatory test to screen dogs, or it can be a useful confirmatory test for seropositive dogs (*23*,*24*,*29*). However, use of PCR is not yet readily available in most diagnostic laboratories and remains an experimental test.

## *B. canis* Infection in Humans

Humans acquire *B. canis* infection through direct contact with infected dogs or their reproductive or blood products (*30*–*32*). Clinical signs and symptoms include undulant fever, chills, malaise, splenomegaly, and peripheral lymphadenomegaly (*33*). In humans, diagnosis is often complicated because of the nonspecific signs and symptoms coupled with a low index of suspicion by many physicians. If the disease is part of the differential diagnosis, culture is the only test available for diagnosing *B. canis* infection in humans, and confirmation is problematic because of low-level and intermittent bacteremia (*34*). Even if physicians suspect brucellosis, diagnoses may be missed because the commercially available serologic tests screen for the smooth *Brucella* species and will not detect antibodies against *B. canis* (*35*). Canine serologic tests for *B. canis* infection have been adapted for use in humans, but test results should be interpreted with caution.

Laboratory personnel, veterinarians, and animal caretakers are at increased risk for exposure to *B. canis* (*3*,*32*,*36*). *Brucella* spp. are considered high-risk pathogens and require a specialized Biosafety Level 3 work space, which if not used can result in laboratory-acquired exposure from a variety of scenarios, such as working with unknown bacterial pathogens on the benchtop (*28*). Dentinger et al. described an incident in which 31 laboratory workers were exposed to *B. canis* after handling an unknown gram-negative bacterium on the benchtop (*16*). None became ill with clinical disease, even those characterized as having experienced high-risk exposures (according to Centers for Disease Control and Prevention guidelines) and who declined postexposure prophylaxis (5 of 21 at high risk) (*16*). One case of laboratory-acquired exposure was documented in a technician who used mouth-pipetting to resuspend the M– strain of *B. canis*; the technician experienced symptoms despite this particular strain being considered avirulent in dogs (*37*). Additionally, Krueger et al. applied available veterinary serologic diagnostic tests to 2 cohorts of persons with or without occupational exposure to dogs and found a seroprevalence of 3.6% among those exposed to dogs, which is higher than previously reported seroprevalence of 0.6% among those with occupational exposure (*3*,*38*). Identified risk factors included working as kennel staff, exposure to breeding bitches, and failure to wash hands after caring for a sick dog (*3*). Of note, in that study, only 2 of the 306 persons with occupational exposure to dogs reported any clinical signs or symptoms associated with brucellosis after contact with dogs who had confirmed brucellosis (*3*). Unfortunately, the temporality of the onset of clinical signs and symptoms and exposure could not be determined (*3*). Regardless, these findings may suggest that healthy humans might be moderately resistant to clinical illness from *B. canis* infection.

Several case reports highlight pet ownership as a likely risk factor leading to infection in otherwise healthy persons (*9*,*16*,*32*,*33*,*39*). In particular, children and immunosuppressed persons might be at higher risk for acquiring the disease (*16*,*36*,*39*,*40*). Three cases in children <4 years of age have been reported (*16*,*36*,*39*). In 1 of the reports, Dentinger et al. described transmission of *B. canis* to a child from an infected puppy that had been purchased from a pet store and was deemed healthy during an initial veterinary visit (*16*). However, after the child became febrile and *B. canis* infection was diagnosed by blood culture, isolates from the child and puppy were submitted to the Centers for Disease Control and Prevention. The 2 isolates showed close genetic similarity, suggesting that the puppy was the source of infection. Clinical signs did not develop in 4 adults in the same household, all of whom had been exposed to the puppy. Several recent reports of *B. canis* in HIV-infected patients highlight the risk within this population (*31*,*40*,*41*). These cases of *B. canis* infection were linked to ownership of reproductively intact dogs that had a history of reproductive failure and a later diagnosis of *B. canis* infection according to serology and blood culture (*31*,*40*).

## Public Health Implications

Brucellosis in dogs occurs worldwide (Figure), but many countries, regardless of their resource level, lack a cohesive plan to respond to cases of this infection in humans or dogs. Brucellosis in humans is notifiable in all 57 states and territories of the United States. Thus, cases must be reported to the National Notifiable Disease Surveillance System; reported in a case report to the Bacterial Special Pathogens Branch at the Centers for Disease Control and Prevention when identified by a health provider, hospital, or laboratory; or both. However, the causative *Brucella* species is not always reported. As a result, it is difficult to obtain accurate estimates of *B. canis* infections in humans. Despite the presence of this pathogen in geographically and politically diverse locations, few countries have *B. canis*–specific regulations. A lack of regulatory interest makes it likely that *B. canis* will continue to be an underrecognized pathogen of dogs and humans.

The public health relevance of *B. canis* infection in humans is unclear because much of the information comes from case reports. The perceived infrequency of human infection with *B. canis* and the lack of reliable diagnostic tools for disease detection has led to few serologic surveys in humans. Our current understanding of prevalence of *B. canis* infection in humans comes from a handful of serologic surveys that use diagnostic tests available for dogs and thus may not be truly representative (*3*,*38*,*42*–*44*).

In the United States, cross-sectional serologic surveys of military recruits and Florida residents and case–control surveys of animal caretakers with occupational exposure to canids documented an extremely low *B. canis* seropositivity (0.4%–0.6%) (*38*,*42*,*44*). Veterinarians from Florida with occupational exposure to dogs were also surveyed but were all negative according to serologic testing (*38*). In 1976, a serologic survey in Mexico City, Mexico, evaluated human blood samples from randomly selected patients for *B. canis* antibodies by using the plate agglutination test; documented seropositivity was 13.3% (*45*). More recently, in Brazil, convenience sampling of human blood samples for screening found that 4.6% of surveyed adults had a positive antibody titer (*46*). Most serologic studies have relied on random convenience sampling of human blood samples. In contrast, a case–control survey by Monroe et al. documented a high *B. canis* seropositivity (80.5%) in persons with fever of unknown origin, but these results were not confirmed by blood culture (*43*). Differences between these studies can be attributed to the test used (tube agglutination test vs. microtiter plate agglutination) and the study population.

When compared with owned dogs, stray dogs are more likely to be intact and have a higher documented level of *B. canis* seropositivity (*45*,*47*). A higher burden of canine brucellosis in the stray/roaming dog populations could lead to spillover into the human population in areas with a large number of intact, stray dogs because these dogs are taken into shelters or placed in foster homes pending adoption. In the United States, ≈30% of pet dogs are adopted from animal shelters, and testing for *B. canis* is not standard procedure before adoption (*48*). No definitive evidence demonstrates a direct link between the number of reproductively intact, stray dogs in an area and potential for human exposure. Studies that attempt to compare levels of *B. canis* antibodies in humans with results of serologic surveys of dogs may not correlate a positive antibody titer in humans to clinical signs of infection or may not correlate the findings with exposure to stray or owned dogs (*45*). In the absence of the full epidemiologic picture, it is difficult to draw conclusions between seropositive dogs and the potential for human exposure, but future research could clarify the risk potential.

Another potential source of *B. canis* dissemination is breeding kennels, given the nature of the disease, the fact that animals are housed in close contact, and the constant movement of dogs for breeding or sale (*49*). Recent outbreaks in kennels in the United States, Hungary, Sweden, and Colombia highlight the link between outbreaks and interregional/international movement of breeding dogs (*5*–*7*,*49*). Unrestricted movement of reproductively intact dogs or puppies is a known risk factor for the spread of infectious diseases and has led to human infection with *B. canis* (*16*,*49*). Quarantine periods and premovement health tests of dogs vary by region, but no region tests dogs for brucellosis before they are moved (*48*). Required testing of breeding animals or their offspring before interstate or international movement would decrease the risk for *B. canis* transmission between dogs and from dogs to humans.

Practices to limit the number of intact stray animals include government- or private charity–sponsored sterilization or testing and euthanasia of *B. canis*–positive dogs. In resource-limited communities, the true risk associated with a large roaming population is unknown, but these dogs should be considered a possible zoonotic risk for humans until new data suggest otherwise. This population of dogs serves to keep brucellosis as an endemic zoonotic disease indefinitely.

The World Health Organization and the World Organisation for Animal Health do not have policies relating to brucellosis caused by *B. canis*. Perhaps because of a perceived low incidence, many countries also do not have response plans or routine surveillance for *B. canis* in dogs or humans (*5*,*46*). In the United States, where *B. canis* was first isolated, the response is piecemeal; however, published recommendations include requiring mandatory reporting of brucellosis in dogs to state health authorities, state health departments to enter into a memorandum of understanding with veterinary diagnostic laboratories to report positive cases to the state health department, and mandatory communication with veterinarians and dog owners to alert them of the zoonotic risk (*30*). Other measures to prevent zoonotic transmission include confirming the diagnosis with the veterinarian and providing educational materials about the zoonotic potential associated with interacting with a *B. canis*–positive dog (*30*). One aspect of reducing the zoonotic potential is educating owners about options for managing *B. canis*–positive dogs, such as sterilization, antimicrobial drug therapy, and repeat testing, or euthanasia if those measures cannot be applied (*30*). Anyone who has contact with an infected dog should maintain good hygiene standards when handling its urine, feces, or reproductive products (*30*).

Other methods to decrease the incidence of brucellosis in dogs include improving diagnostic tests and developing a vaccine. Improved diagnostic tests are needed for better evaluation of disease prevalence in at-risk communities and to help physicians and veterinarians more accurately identify cases of disease caused by *B. canis*. In addition to improved diagnostic tests, a *B. canis* vaccine, which is not currently available, could substantially decrease infection incidence in the dog population and thus reduce the risk for transmission to humans.

In conclusion, brucellosis in dogs remains endemic to many parts of the world and without stronger intervention measures will probably remain an underrecognized threat to human health and animal welfare. Future work is required to improve diagnostic assays for humans and animals and to generate policies to prevent the spread of disease. Implementation of mandatory testing before interstate or international movement of dogs would be a good first step.

Technical Appendix Serologic surveys for brucellosis in dogs.
